# Editorial: Advances in understanding the interplay of soil carbon, iron, and arsenic transformation

**DOI:** 10.3389/fmicb.2025.1697359

**Published:** 2025-09-26

**Authors:** Zhao-Feng Yuan, Xiangfeng Tan, Weiwei Zhai, Williamson Gustave

**Affiliations:** ^1^State Key Laboratory for Quality and Safety of Agro-Products, Key Laboratory of Biotechnology in Plant Protection of MARA, Zhejiang Key Laboratory of Green Plant Protection, Institute of Plant Virology, Ningbo University, Ningbo, China; ^2^International Science and Technology Cooperation Base for the Regulation of Soil Biological Functions and One Health of Zhejiang Province, Ningbo University, Ningbo, China; ^3^Institute of Digital Agriculture, Zhejiang Academy of Agricultural Sciences, Hangzhou, China; ^4^Zhejiang Key Laboratory of Low-carbon Control Technology for Industrial Pollution, College of Environment, Zhejiang University of Technology, Hangzhou, China; ^5^School of Chemistry, Environmental and Life Sciences, University of the Bahamas, New Providence, Nassau, Bahamas

**Keywords:** soil, biotic process, regulation, carbon, iron, arsenic

Soils are critical regulators of elemental cycling, mediating interactions among carbon (C), iron (Fe), and arsenic (As) that influence ecosystem function, climate regulation, and environmental health. Soil organic carbon (SOC) represents the largest terrestrial carbon reservoir and is often considered a natural solution for mitigating climate change. However, the stability of SOC is strongly mediated by its interactions with Fe oxides and hydroxides, which can either protect organic matter through mineral associations or promote its loss under reducing conditions (Xu and Tsang, 2024; Hu et al., 2025). The transformation of Fe, in turn, directly governs the fate of As, one of the most hazardous environmental contaminants, through adsorption, reduction, and microbial methylation pathways (Gao et al., 2024; Tang et al., 2024).

The complex interplay of these processes highlights the need for integrative approaches that couple soil chemistry, microbial ecology, and environmental engineering. While Fe minerals provide protective surfaces that stabilize SOC, their reductive dissolution can release both C and As into more labile pools, with microorganisms playing central roles in mediating redox transformations (Yao et al., 2023; Wang et al., 2024a). Horizontal gene transfer and viral interactions further add layers of complexity (Wang et al., 2024b; Liang et al., 2025). Yet, despite growing evidence for these interconnections, major knowledge gaps remain, particularly in predicting the dynamics of C–Fe–As coupling under fluctuating redox conditions and in understanding the roles of less-studied microbial groups.

To address these challenges, this Research Topic brings together six contributions spanning mechanistic, methodological, and applied perspectives. Collectively, they shed light on the stabilizing functions of minerals, the roles of microbes in soil health and disease, the use of engineered amendments and bioreporters, and innovative bioelectrochemical approaches to pollution management.

One set of studies focuses on the mineralogical and structural controls of soil processes. Li and Guo revealed how soil microaggregates vary along an elevation gradient in Tongbai Mountain, with Mn- and Fe-rich microaggregates at low elevations promoting metal mobility, while high elevations favored the formation of organo-mineral complexes that stabilized C, N, and Fe. This highlights how landscape position can mediate elemental coupling and provides a framework for anticipating how mountain soils may respond to climate change. In parallel, Chen et al. explored engineered soil amendments, showing that silicon–iron modified biochars effectively reduced the bioavailability of Cd and As in paddy soils by altering speciation pathways. Their work emphasizes the potential for Fe- and Si-based additives to immobilize contaminants while simultaneously influencing microbial functional genes involved in As oxidation and Cd precipitation. Together, these studies highlight the central role of Fe mineral phases, whether natural or engineered, in regulating C–Fe–As transformations and contaminant dynamics.

A second theme centers on microbial tools and ecological perspectives. Zhang R. et al. provided an opinion on whole-cell bioreporter (WCBs) technology as an emerging tool for assessing As risk in soils. Unlike traditional chemical assays, WCBs can differentiate As species and measure bioavailable fractions, offering ecologically relevant insights into toxicity. Complementing this, Tong et al. reviewed the persistence and pathogenicity of *Fusarium oxysporum* in watermelon soils. Although focused on plant pathology, their review illustrates how soil microbial communities and environmental factors interact to sustain long-term pathogen survival, mirroring the challenges of predicting microbial mediation in C–Fe–As cycles. Both contributions underscore the value of microbial systems, whether as tools or as agents, for monitoring and managing soil processes.

Technological innovation emerges as another unifying thread. Zhang X. et al. synthesized over 10,000 cases of microbial fuel cells' (MFCs) studies to identify the strongest drivers of performance. Their analysis showed that cathode chamber volume and surface area are key predictors of power density, while biological pretreatment of substrates significantly enhances efficiency. Importantly, MFCs are not only promising for energy generation but also for pollutant removal, including heavy metals and organics. Meanwhile, Tian et al. identified a new soilborne pathogen, *Ilyonectria robusta*, causing basal stem rot in *Schisandra chinensis*. Their study demonstrates how integrating molecular tools with field surveys can rapidly identify emerging risks to soil–plant systems, further reinforcing the importance of innovation in soil biogeochemistry and health research.

Collectively, these contributions illustrate the interconnectedness of mineral, microbial, and technological dimensions in advancing soil science. Mineral studies demonstrate how natural and engineered Fe associations mediate contaminant dynamics; microbial perspectives highlight the dual roles of soil organisms as both sentinels and stressors; and technological innovations, from MFCs to WCBs, open new pathways for monitoring and remediation. Together, they provide a multifaceted view of the challenges and opportunities in managing C–Fe–As interactions in soils. While the studies in this Research Topic significantly broaden our understanding of soil biogeochemistry, they also point to critical areas for future research. There is a pressing need to integrate mineralogical, microbial, and engineering perspectives into predictive frameworks that can capture soil heterogeneity and dynamic redox processes. Future work should explore the underappreciated roles of viruses, archaea, fungi, and microplastics in shaping elemental cycles, as well as the feedbacks between soil processes and climate drivers. Scaling laboratory findings to field applications will remain a central challenge, requiring interdisciplinary approaches that link soil biogeochemistry to agronomy, hydrology, and environmental engineering.

## References

[B1] Gao M. Li H. Xie Z. Li Z. Luo Z. Yu R.. (2024). The fate of arsenic associated with the transformation of iron oxides in soils: the mineralogical evidence. Sci. Total Environ. 914:169795. 10.1016/j.scitotenv.2023.169795 38199364

[B2] Hu S. Zhang H. Yang Y. Wang P. Ding Z. Chen G.. (2025). Organic carbon sequestration by secondary Fe–Mn complex minerals via the anoxic redox reaction of Fe (II) and birnessite. Environ. Sci. Technol. 59, 15128–15141. 10.1021/acs.est.4c12756 40632648

[B3] Liang X. Yang S. Radosevich M. Wang Y. Duan N. Jia Y. (2025). Bacteriophage-driven microbial phenotypic heterogeneity: ecological and biogeochemical importance. NPJ Biofilms Microbiomes 11:82. 10.1038/s41522-025-00727-5 40399330 PMC12095545

[B4] Tang H. Xiang G. Xiao W. Yang Z. Zhao B. (2024). Microbial mediated remediation of heavy metals toxicity: mechanisms and future prospects. Front. Plant Sci. 15:1420408. 10.3389/fpls.2024.1420408 39100088 PMC11294182

[B5] Wang S. Gao W. Ma Z. Zhu Z. Luo Y. Wei L.. (2024a). Iron mineral type controls organic matter stability and priming in paddy soil under anaerobic conditions. Soil Biol. Biochem. 197:109518. 10.1016/j.soilbio.2024.109518

[B6] Wang S. Zhu D. Ge T. Wang Y. Zhang Y. Liang C.. (2024b). Unveiling the top-down control of soil viruses over microbial communities and soil organic carbon cycling: a review. Clim. Smart Agric. 1:100022. 10.1016/j.csag.2024.100022

[B7] Xu Z. Tsang D. C. (2024). Mineral-mediated stability of organic carbon in soil and relevant interaction mechanisms. Eco-Environ. Health 3, 59–76. 10.1016/j.eehl.2023.12.003 38318344 PMC10840363

[B8] Yao Y. Wang L. Peduruhewa J. H. Van Zwieten L. Gong L. Tan B.. (2023). The coupling between iron and carbon and iron reducing bacteria control carbon sequestration in paddy soils. Catena 223:106937. 10.1016/j.catena.2023.106937

